# Systemic DKK1 neutralization enhances human adipose‐derived stem cell mediated bone repair

**DOI:** 10.1002/sctm.20-0293

**Published:** 2020-12-30

**Authors:** Stefano Negri, Yiyun Wang, Takashi Sono, Qizhi Qin, Ginny Ching‐Yun Hsu, Masnsen Cherief, Jiajia Xu, Seungyong Lee, Robert J. Tower, Victoria Yu, Abhi Piplani, Carolyn A. Meyers, Kristen Broderick, Min Lee, Aaron W. James

**Affiliations:** ^1^ Department of Pathology Johns Hopkins University Baltimore Maryland USA; ^2^ Orthopaedic and Trauma Surgery Unit, Department of Surgery, Dentistry, Paediatrics and Gynaecology University of Verona Verona Italy; ^3^ Department of Orthopaedic Surgery Johns Hopkins University Baltimore Maryland USA; ^4^ Department of Plastic Surgery Johns Hopkins University Baltimore Maryland USA; ^5^ School of Dentistry University of California Los Angeles Los Angeles California USA

**Keywords:** adipose stem cell, adipose stromal cell, bone healing, bone repair, bone tissue engineering, mesenchymal stem cell, Wnt signaling

## Abstract

Progenitor cells from adipose tissue are able to induce bone repair; however, inconsistent or unreliable efficacy has been reported across preclinical and clinical studies. Soluble inhibitory factors, such as the secreted Wnt signaling antagonists Dickkopf‐1 (DKK1), are expressed to variable degrees in human adipose‐derived stem cells (ASCs), and may represent a targetable “molecular brake” on ASC mediated bone repair. Here, anti‐DKK1 neutralizing antibodies were observed to increase the osteogenic differentiation of human ASCs in vitro, accompanied by increased canonical Wnt signaling. Human ASCs were next engrafted into a femoral segmental bone defect in NOD‐*Scid* mice, with animals subsequently treated with systemic anti‐DKK1 or isotype control during the repair process. Human ASCs alone induced significant but modest bone repair. However, systemic anti‐DKK1 induced an increase in human ASC engraftment and survival, an increase in vascular ingrowth, and ultimately improved bone repair outcomes. In summary, anti‐DKK1 can be used as a method to augment cell‐mediated bone regeneration, and could be particularly valuable in the contexts of impaired bone healing such as osteoporotic bone repair.

## Graphical abstract

Systemic anti‐DKK1 treatment promotes engrafted stem cell survival, induces vascular ingrowth, and catalyzes bone matrix deposition among human adipose stem cell (ASC) treated femoral segmental bone defects. BM, bone marrow; CB, cortical bone; S, scaffold.

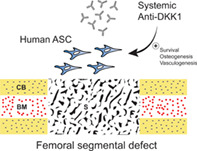

**Significance statement**

Mesenchymal stem/stromal cell‐mediated bone repair shows promise, yet inconsistent and incomplete tissue regeneration has been a persistent challenge. Here, systemic anti‐DKK1 treatment improves upon progenitor cell mediated bone repair outcomes in a preclinical xenograft model. In the future, DKK1 may represent a targetable molecular “brake” on the process of stem cell mediated bone formation, and release of this brake via neutralizing antibodies could be a method to improve bone repair outcomes.

## 1 INTRODUCTION

Nonhealing skeletal defects are addressed in millions of surgeries worldwide each year, in diverse fields such as orthopedic, neurocranial, plastic, and oral and dental surgery. Segmental bone defects can be the result of congenital abnormalities, or can arise secondarily from diverse causes such as trauma, malignancy, or infection. In adult patients, a critical defect of long bones is defined as a bone loss involving >50% of the circumference or >2 cm in length.^1^ The available techniques to surgical manage these conditions are mainly based on bone grafting, distraction osteogenesis, and the induced membrane technique.^2^ These approaches require long times of recovery and multiple surgeries.^3^ The growing biomedical burden of skeletal defects coupled with the lack of adequate treatment options has fueled the interest in alternative therapies for bone regeneration, especially in the osteoporotic patient.

Adipose‐derived stem cells (ASCs) have been used extensively to induce bone repair.^4^, ^5^ Although many experiences report a good potential of ASCs in bone healing,^6^, ^7^, ^8^ batch‐to‐batch variability and cellular heterogeinity have been identified.^7^, ^9^, ^10^ Indeed, contaminant nonprogenitor cells have been found to represent an obstacle to the osteogenic efficacy of ASC in several contexts.^11^, ^12^, ^13^ Indeed, recent studies have observed that without additional biological augments, ASCs may have limited application in bone tissue engineering.^14^, ^15^ To circumvent this issue, stem/progenitor cell purification by cell sorting techniques has been published by our group and others.^9^, ^16^, ^17^ However, the level of complexity for cell isolation is high, leading to regulatory challenges in clinical translation. A simpler solution would be to pharmacologically target those signaling pathways expressed in unpurified stromal cell population that may inhibit the process of osteogenic differentiation.

Dickkopf‐1 (DKK1) is an extracellular Wnt antagonist regulating bone formation. Its impact on bone physiology is by competing with Wnt ligands for binding to coreceptors lipoprotein‐related proteins 5 and 6 (LRP5 and LRP6).^18^ Several studies have shown that DKK1 neutralizing antibodies (anti‐DKK1) can accelerate bone formation and increase bone mineral density (BMD) in various animal models.^19^ Systemic anti‐DKK1 therapy has shown improved fracture healing capacity in rodent long bone fracture models.^20^, ^21^, ^22^ The overall safety profile of anti‐DKK1 has been confirmed in several preclinical models as well as in human clinical trials.^23^, ^24^, ^25^ Recently, our laboratory reported that DKK1 is highly expressed in human ASCs, and anti‐DKK1 improves the early osteogenic differentiation of human ASC in vitro.^26^ Despite this accumulating translational evidence, the combination of anti‐DKK1 with a stem/stromal cell therapy has not been examined in the context of in vivo bone repair.

In this study, anti‐DKK1 treatment was examined as a means to improve outcomes associated with ASC mediated bone defect repair. In order to assess this, human ASCs were engrafted into femoral segmental bone defect in NOD‐*Scid* mice, with animals subsequently systemically treated with either anti‐DKK1 or isotype control during the repair process. Overall, systemic anti‐DKK1 induced an increase in human ASC engraftment and survival, an increase in vascular ingrowth, and ultimately improved bone repair outcomes.

## 2 MATERIALS AND METHODS

### 2.1 Isolation of human ASCs from adipose tissue

Liposuction was obtained from a healthy adult donor, under Institutional Review Board (IRB) approval (protocol number IRB00119905) and a waiver informed consent. Liposuction was stored at 4°C and processed within 48 hours. ASCs were obtained according to the previously published method.^9^, ^14^, ^27^, ^28^ Equal volume phosphate‐buffered saline (PBS) was used to wash the lipoaspirate. Washed liposuction was digested at 37°C for 60 minutes with 1 mg/mL collagenase II in Dulbecco modified Eagle medium (DMEM) containing 3.5% bovine serum albumin (Sigma‐Aldrich, St. Louis, Missouri) under agitation. After centrifugation, supernatants containing adipocytes were removed. Meanwhile, the cell pellet was resuspended and incubated in red blood cell lysis buffer (155 mM NH_4_Cl, 10 mM KHCO_3_, and 0.1 mM ethylenediaminetetraacetic acid [EDTA]) at room temperature (RT) for 10 minutes. Next, after centrifugation, cells were resuspended with PBS and filtered at 40 μm. Cells were cultured at 37°C in a humidified atmosphere containing 95% air and 5% CO_2_ and with the standard growth medium consisted of DMEM (Gibco, Grand Island, New York), 10% fetal bovine serum (FBS) (Gibco), 1% penicillin/streptomycin (Gibco), and 2 mg/mL human basic fibroblast growth factor (R&D System, Minneapolis, Minnesota).

### 2.2 Osteogenic differentiation

Osteogenic differentiation medium consisted of DMEM, 10% FBS, 1% penicillin/streptomycin with 100 nM dexamethasone, 10 mM β‐glycerophosphate, and 50 μM ascorbic acid (Sigma‐Aldrich). Cells were cultured with osteogenic differentiation medium containing anti‐DKK1 antibody or IgG isotype control. See Table S1 for antibody information. Medium was changed every 3 days. Alizarin red S (Sigma‐Aldrich) staining was used to detect mineralization. Sodium hydroxide (0.1 N) was used to dissolve the calcium precipitate and quantified by absorbance at 548 nm. Mineralization on hydroxyapatite coated poly(lactic‐*co*‐glycolic acid) (HA‐PLGA) scaffolds was evaluated in ASCs treated with IgG or anti‐DKK1 in osteogenic medium for 24 hours before seeding. Next, the cellular scaffolds were cultured for 7 days and alizarin red staining was performed.

### 2.3 RNA isolation and quantitative real‐time polymerase chain reaction

TRIzol (Life Technology, Waltham, Massachusetts) was used for total RNA isolation. Then, according to the manufacturer's instructions, iScript cDNA Synthesis Kit (Bio‐Rad, Hercules, California) was used to generate cDNA from RNA. SYBR Green PCR Master Mix (Life Technology) was used for quantitative real‐time polymerase chain reaction (qRT‐PCR). Primer information is provided in Table S2. N = 3 wells per group, and all studies were performed in three biological replicates.

### 2.4 Scaffold preparation with ASC

Implants were prepared using 7.5 × 10^5^ total ASC per scaffold. HA‐PLGA scaffolds were custom fabricated using previously published methods^29^ and were cylindrical measuring 3.5 mm in length and 2 mm diameter. For cellular attachment, each scaffold was placed into an individual well of a 24‐well plate. 7.5 × 10^5^ human ASC in 10 μL DMEM medium were seeded onto the scaffold and incubated at 37°C for 10 minutes. Next, the scaffold with cells was wet with an additional 70 μL DMEM medium and incubated at 37°C for 1 hour. Cell‐scaffold interaction was characterized in vitro focusing on distribution and cell viability. Briefly, scaffolds with cells were fixed in 4% paraformaldehyde, embedded in the optimum cutting temperature compound and sectioned at 16 μm thickness. Sections were counterstained with 4′,6‐diamidino‐2‐phenylindole (DAPI) mounting medium (H‐1500, Vector Laboratories, Burlingame, California) and imaged with Leica DM 6B microscope (Leica Biosystems, Germany). Viability was evaluated with live‐dead staining (MilliporeSigma, Burlington, Massachusetts)^30^ after 1 hour, and 7 days after treatment with either anti‐DKK1 or IgG. Scanning electron microscopy (SEM) was used to confirm the attachment of cells to the scaffold. Briefly, samples were fixed in 2.5% glutaraldehyde, 3 mM MgCl2, in 0.1 M sodium cacodylate buffer, pH 7.2 overnight at 4°C. After buffer rinse, samples were postfixed in 1% osmium tetroxide in 0.1 M sodium cacodylate buffer (1 hour) on ice in the dark. Following a DH_2_O rinse, samples were dehydrated in a graded series of ethanol and left to dry overnight in a desiccator with hexamethyldisilazane. Samples were mounted on carbon‐coated stubs and imaged on the Zeiss Leo field emission scanning electron microscope at 1 kV.

To evaluate the impact of DKK1 on cell adhesion, ASCs were pretreated either with anti‐DKK1 or IgG in osteogenic medium for 24 hours. Then, 7.5 × 10^5^ human ASC in 10 μL DMEM medium were seeded onto the scaffold and incubated at 37°C for 10 minutes. Each scaffold was placed into an individual well of a 48‐well plate with 100 μL DMEM medium and incubated at 37°C for 6 hours. The unattached cells were then quantified.

### 2.5 Animals and conditions

Twelve‐week‐old NOD‐*Scid* male mice were used (strain code 001303, The Jackson Laboratories, Bar Harbor, Maine). Experimental procedures were consistent with ethical principles for animal research and were approved by Johns Hopkins University ACUC (protocol number MO18M144). Throughout the study, mice were housed in an IVC system rack using polypropylene cages (19 cm × 28 cm × 13 cm), with 12/12 night/day cycles, 21°C (±2°C) and 50% (±20%) relative humidity. All mice had ad libitum access to complete mouse food and filtered water. Animal allocation is described in Table S3.

### 2.6 Surgical procedure

A 3.5‐mm mid‐diaphyseal femoral segmental defect (FSD) was created and stabilized by plate osteosynthesis as previously described.^31^ To perform the skeletal defect, animals were anesthetized with inhaled isoflurane (3%‐5% induction, 2%‐3% maintenance) delivered with combined oxygen and nitrous oxide (1:2 ratio) along with subdermal injection of sustained‐release buprenorphine (1.2 mg/kg subcutaneous, q72h). Briefly, a 18 to 20 mm skin incision on the lateral aspect of the thigh. After the incision of the fascia lata, the interval between the vastus lateralis and biceps femoris muscles was identified and using a smooth periosteal elevator (Roboz Surgical Instrument Co., Maryland) the femoral diaphysis exposed. A 6‐hole polyether ether ketone (PEEK) micro‐locking plate (MouseFix, 10 mm long; 1.5 mm wide, RISystem AG, Switzerland) was positioned on the anterior femoral side and after drilling the respective holes with a 0.30 mm diameter perforator operated by a miniature electrical pen drill (RISystem AG), the proximal two and distal screws were inserted using the dedicated holder. The osteotomy was performed using a 10 mm diameter, 0.15 mm thick diamond rotary saw (Henry Schein, Inc., Melville, New York) under continuous saline irrigation. A metal osteotomy guide (RISystem GA) was used to perform two precise osteotomies at predetermined 3.5 mm distances on each side of the defect. Segmental defects were then treated by the insertion of a custom fabricated HA‐coated PLGA scaffold. The overlying intermuscular interval was closed with 4‐0 absorbable suture (Ethicon, Inc., Somerville, New Jersey) to create a muscular pouch covering the defect. The skin incision was closed with 5‐0 Prolene suture (Ethicon, Inc.). Mice were sacrificed 8 weeks postoperatively via CO_2_ overdose, and the tissue harvested for analysis.

### 2.7 Experimental groups

Mice were divided into four different treatment groups: (a) systemic IgG (15 mg/kg, SC, twice a week for 4 weeks) with acellular scaffold, (b) systemic anti‐DKK1 (15 mg/kg, SC, twice a week for 4 weeks) with acellular scaffold, (c) systemic IgG with ASC laden scaffold (7.5 × 10^5^ ASCs per defect), and (d) systemic anti‐DKK1 with ASC laden scaffold (7.5 × 10^5^ ASCs per defect site). Animal allocation is further summarized in Table S3.

### 2.8 High‐resolution roentgenography, dual‐energy X‐ray absorptiometry, and microcomputed tomography assessments

Bone repair was assessed using a combination of high‐resolution roentgenography (XR), dual‐energy XR absorptiometry (DXA), and microcomputed tomography (μCT) imaging. First, the BMD of the defect site was prospectively analyzed every 4 weeks with DXA using a rectangular region of interest (ROI) centered within the femoral defect (Faxitron Bioptics, Tucson, Arizona). Serial quantification of BMD was also assessed within the lumbar spine, with a ROI encompassing the L1 to L6 vertebral bodies as well as the whole contralateral femur. Second, high‐resolution XR imaging was also performed to survey bone healing every 4 weeks. Third, general morphological appearance and morphometric analysis were performed using ex vivo microCT using a Skyscan 1275 scanner (Bruker‐MicroCT, Kontich, Belgium) with the following settings: 65 kV, 153 μA, 1 mm aluminum filter in 180°, six frames per 0.3° with a 9‐μm voxel size. Images were reconstructed using NRecon. DataViewer software was used to realign the images and quantitative parameters were assessed using Skyscan CTan software (SkyScan, Kontich, Belgium) as previously published.^32^ Briefly, a three‐dimensional (3D) cylindrical ROI of 4.5 mm length and 2.5 mm diameter was set between the inner two screws of the plate. A threshold value range of 800 to 1250 Hounsfield units (HU) was used.^33^, ^34^ After global thresholding was carried out, a 3D data analysis, including bone volume (BV), bone volume/tissue volume (BV/TV), BMD, trabecular thickness (Tb.Th), and trabecular number (Tb.N), was performed.

### 2.9 Quantification of osseous integration

The analysis of the bone‐hardware interface was conducted following the guideline of Bruker for the quantification of bone around a metal implant (Method note MCT‐074). Briefly, the plane of analysis was oriented orthogonally to the main axis of each screw. A volume of interest (VOI) mask was created based on the binary of the metal screw. This binary is filled and dilated to create an ROI surface at a constant distance from the metal surface, set by the pixel value of the dilation. In this manner, a hollow ring around the implant of 20 pixels thickness was obtained. After global thresholding, the BV of this ROI was measured using a 3D data analysis. All of the implanted screws of each animal were analyzed, and values reported as a mean value per animal.

### 2.10 Histology and immunohistochemistry

Specimens were harvest and fixed in 4% paraformaldehyde for 24 hours. Fourteen percentage of EDTA was used to decalcify the specimens for 1 month. Next, specimens were embedded in O.C.T compound (Tissue‐Tek Sakura Finetek USA, Inc., California), and sectioned at 16 μm thickness. H&E, modified Goldner's Trichrome (GMT), Safranin O/Fast green (SO/FG), and Tartrate‐resistant acid phosphatase (TRAP) staining were performed on the sections. For immunofluorescent immunohistochemical staining, sections were blocked with 5% goat serum in PBS for 1 hour at RT and incubated with primary antibodies overnight at 4°C. See Table S2 for antibody information. Next, Alexa Fluor 647 or DyLight 594‐conjugated secondary antibodies (1:200) were used. Finally, sections were counterstained with DAPI mounting medium (H‐1500, Vector laboratories). A Leica DM 6B microscope (Leica Biosystems) was used to obtain images.

### 2.11 Statistical analysis

Results are expressed as the mean ± SD. Following an *F* test of the homogeneity of the variances, a Student's *t* test was used for two‐sample comparisons. A one‐way analysis of variance (ANOVA) with Tukey's multiple comparisons test was used for more than two group comparisons (Graphpad Software 8.1). *P < .05 and **P < .01 were considered significant.

## 3 RESULTS

### 3.1 Anti‐DKK1 enhances human adipose derived stem/stromal cell (ASC) osteogenic differentiation via Wnt signaling disinhibition

**FIGURE 1:**
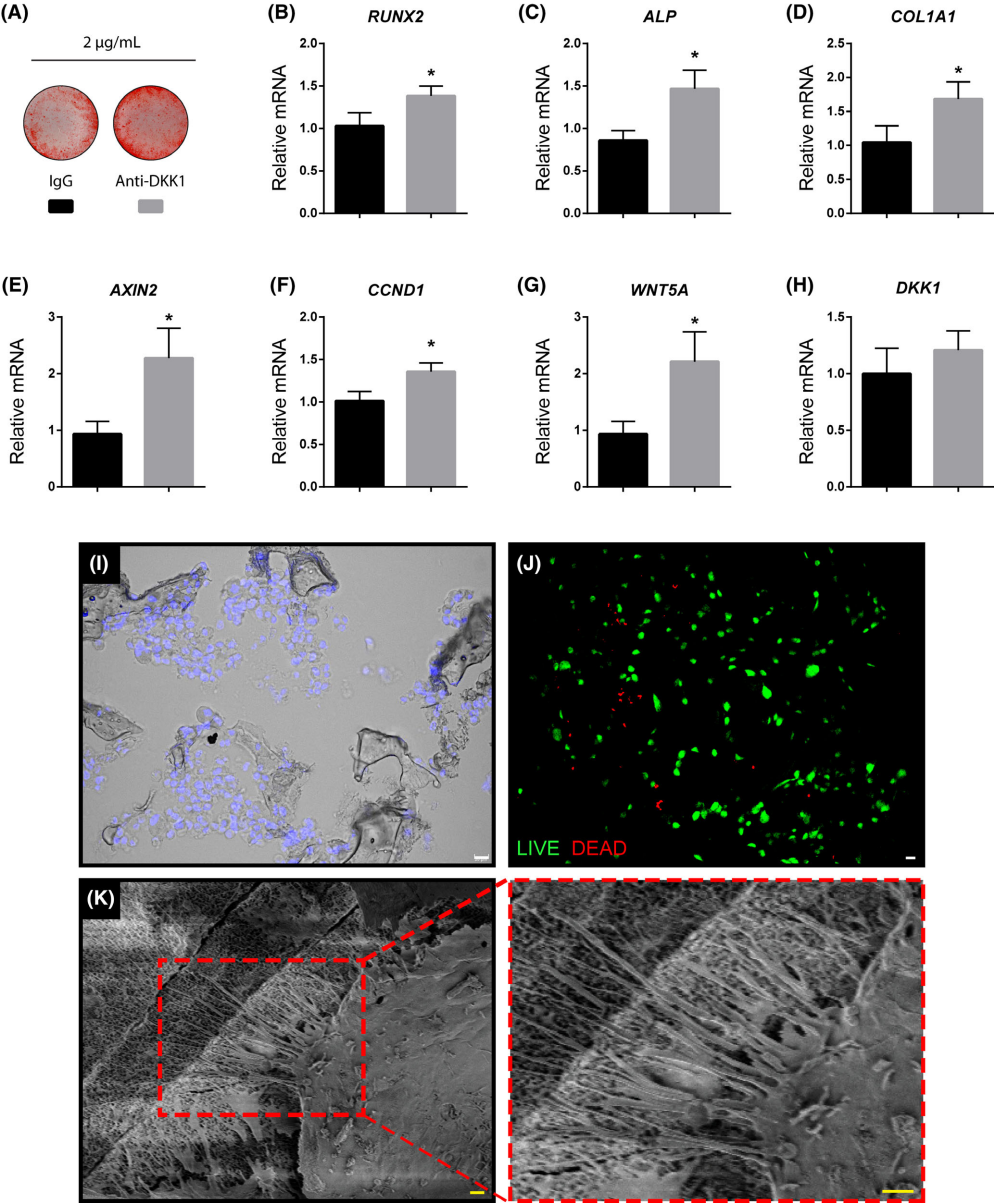
In vitro validation of anti‐DKK1 treatment and viability of cultured human ASCs after seeding on a composite osteoconductive scaffold. A‐D, Effects of anti‐DKK1 on human adipose derived stem/stromal cell (ASC) osteogenic differentiation. A, Alizarin red staining after 7 days of osteogenic differentiation (2 μg/mL anti‐DKK1 or IgG isotype control). B‐D, Gene expression after 3 days of anti‐DKK1 treatment during osteogenic induction, including (B) *RUNX2* (Runt related transcription factor 2), (C) *ALP* (alkaline phosphatase), and (D) *COL1A1* (Collagen Type I Alpha 1). E‐H, Wnt signaling gene expression with anti‐DKK1 treatment for 3 days, including (E) *AXIN2* (Axis Inhibition Protein 2), (F) *CCND1* (Cyclin D1), (G) *WNT5A* (Wnt Family Member 5A), and (H) *DKK1* (Dickkopf‐1). I‐K, In vitro validation of cell seeding on HA‐PLGA [hydroxyapatite coated poly(lactic‐co‐glycolic acid)] composite scaffolds. I, Distribution of DAPI labeled ASCs (appearing blue) on seeded scaffolds after 1 hour. Differential interference contrast used. J, Live‐dead staining of ASC seeded scaffolds after 1 hour. Live cells appear green while dead cells are red. K, Adhesion of seeded ASCs to the scaffold confirmed with scanning electron microscopy. K′, High power of cell membrane filopodia interacting with the scaffold surface. All experiments were performed with an appropriate isotype IgG control and in at least experimental and biological triplicate. Error bars represent 1 SD. White scale bars = 20 μm; yellow scale bars = 2 μm. *P < .01. ASCs, adipose‐derived stem cells

Effects of anti‐DKK1 on in vitro ASC osteogenic differentiation were first assessed. Consistent with our recent report,^26^ anti‐DKK1 treatment (2 μg/mL) promoted bone nodule deposition by alizarin red staining in comparison to IgG isotype control (Figure 1A). To confirm and expand on this finding, we next assessed the changes in osteogenic gene expression by quantitative real‐time polymerase chain reaction after 3 days of osteogenic differentiation (Figure 1B‐D). Results showed that the levels of *RUNX2* (Runt‐related transcription factor 2), *ALP* (alkaline phosphatase), and *COL1A1* (type I collagen) transcripts significantly increased with anti‐DKK1 treatment in comparison IgG control (increase of 34%, 71%, and 61%, respectively, *P < .01). Next, we assessed gene expression of markers indicative of overall canonical Wnt signaling activity, including *AXIN2* (Axis Inhibition Protein 2) and *CCND1* (Cyclin D1) (Figure 1E‐G). Confirming bioactivity of anti‐DKK1, all gene transcripts were more highly expressed among anti‐DKK1 treated cells in comparison to isotype control (~140% increase in *AXIN2* and *WNT5A*, 34% in *CCND1* transcripts, respectively, *P < .01). No significant change in *DKK1* transcripts was found with anti‐DKK1 treatment (Figure 1H). Thus, and consistent with our prior observations,^26^ anti‐DKK1 enhances the osteogenic differentiation of human ASCs.

### 3.2 Validation of cell seeding on a composite osteoinductive scaffold

Our approach was to improve upon ASC mediated regeneration of a long bone critical defect by the supplementation of anti‐DKK1. In order to deliver human ASCs, we relied on a previously validated, osteoinductive, osteoconductive hydroxyapatite (HÁ)‐coated poly(lactic‐co‐glycolic acid) (PLGA) composite scaffold.^16^, ^29^ Here, cylindrical HA‐PLGA scaffolds were custom fabricated to fit a mouse femoral segmental defect (3.5 mm height, 2 mm diameter). ASC distribution, viability, and attachment onto scaffolds were next assessed (Figure 1I‐K). A homogenous distribution of cells throughout the scaffolds was observed, as shown by seeded scaffolds sectioned and stained with DAPI nuclear counterstaining (Figure 1I). As shown by live‐dead staining, high viability was observed at 1 hour after cell seeding (Figure 1J), which was similar across treatment groups (Figure S1). Cell attachment was observed by SEM, in which after 1 hour seeded ASCs had flattened and spread across the porous surface of the scaffold, with filopodial extensions observed (Figure 1K).

Anti‐DKK1 did not significantly affect cell attachment, observed at 6 hours postseeding (Figure S2). Contrarily, after 7 days of culture in osteogenic differentiation medium on HA‐PLGA scaffolds, ASCs treated with anti‐DKK1 showed increased mineral deposition (29.2% increase in comparison to IgG control; Figure S3).

Having further validated cell seeding protocols, we next sought to determine if anti‐DKK1 treatment could improve upon ASC‐mediated bone repair.

### 3.3 Anti‐DKK1 treatment enhances ASC‐mediated bone formation in a critical size FSD

In our experimental study, four groups were compared within a FSD model in NOD‐*Scid* mice. Treatment groups included: (a) acellular scaffold implantation with systemic IgG isotype control treatment or “IgG” (15 mg/kg, SC, twice weekly), (b) acellular scaffold implantation with systemic anti‐DKK1 treatment or “anti‐DKK1” (15 mg/kg, SC, twice weekly), (c) human ASC‐laden scaffold with systemic IgG isotype treatment or “IgG + ASCs,” and (d) human ASC‐laden scaffold with systemic anti‐DKK1 treatment or “Anti‐DKK1 + ASCs.” See Table S3 for a further description of treatment groups and cell numbers.

**FIGURE 2:**
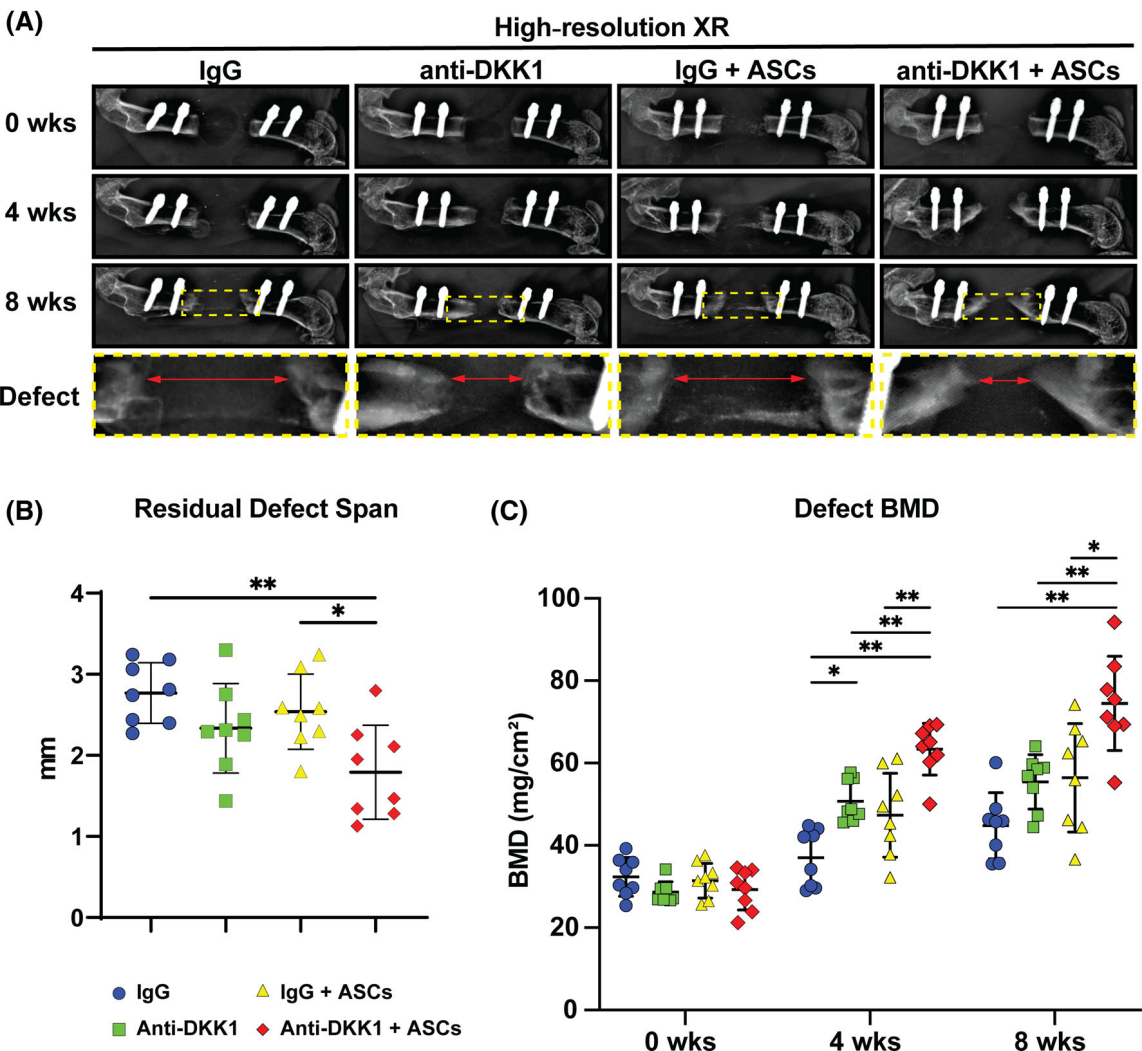
Anti‐DKK1 treatment enhances ASC‐mediated bone formation in a critical size femoral segmental defect. Defects were treated with ASC seeded scaffolds or acellular control scaffolds. Animals were treated with anti‐DKK1 or IgG control (15 mg/kg, SC, twice weekly). A, High‐resolution roentgenography (XR), immediately postoperatively (0 week, first row), at 4 weeks postoperative (second row), 8 weeks postoperative (third row), as well as high magnification of the defect area (bottom row). Each column shows the representative appearance of a treatment group. B, Residual defect span measured on plain films at 8 weeks after surgery. C, Bone mineral density (BMD) of the defect site, as determined by dual‐energy x‐ray absorptiometry at 0, 4, and 8 weeks postoperative. Graphs represent mean and error bars represent 1 SD. See Table S3 for a further summary of animal allocation, treatment regimens, and total cell numbers. *P < .05; **P < .01. ASCs, adipose‐derived stem cells

Defect sites were surveilled by XR every 4 weeks postoperatively (Figure 2A). A qualitative increase in bone growth at the proximal and distal osteotomy sites was appreciated with systemic anti‐DKK1 treatment alone or ASC cell therapy in comparison to IgG control. Bone healing was qualitatively most apparent in ASC treatment groups also provided anti‐DKK1, in which the bone defect edges approximated one another (far right, Figure 2A). After 8 weeks, this qualitative change was confirmed measuring the length of the residual defect span using XR images of each animal (Figure 2B). Combination treatment Anti‐DKK1 + ASCs showed a significant reduction in residual defect span, while either cell therapy or anti‐DKK1 alone showed a nonsignificant trend toward reduction in defect span (Figure 2B). The defect site was further analyzed using DXA every 4 weeks postoperatively (Figure 2C). Here, a gradual increase in BMD was observed across the 8 week time period in all treatment groups. ASC treated defects with anti‐DKK1 treatment showed a significant increase in BMD in comparison to other groups (66% increase in comparison to IgG control, 34% increase in comparison to anti‐DKK1 alone, and 32% increase in comparison to ASC treated defects with IgG control). In comparison, anti‐DKK1 did not increase the BMD at uninjured sites, assessed using both the contralateral femur and lumbar vertebrae (Table S4), a finding consistent with previously published reports in young mice.^35^ In summary, systemic anti‐DKK1 improved ASC mediated FSD healing, as shown by increased BMD, and reduced size of the osteoectomy site.

### 3.4 Anti‐DKK1 enhances ASC‐mediated FSD repair by μCT metrics

**FIGURE 3:**
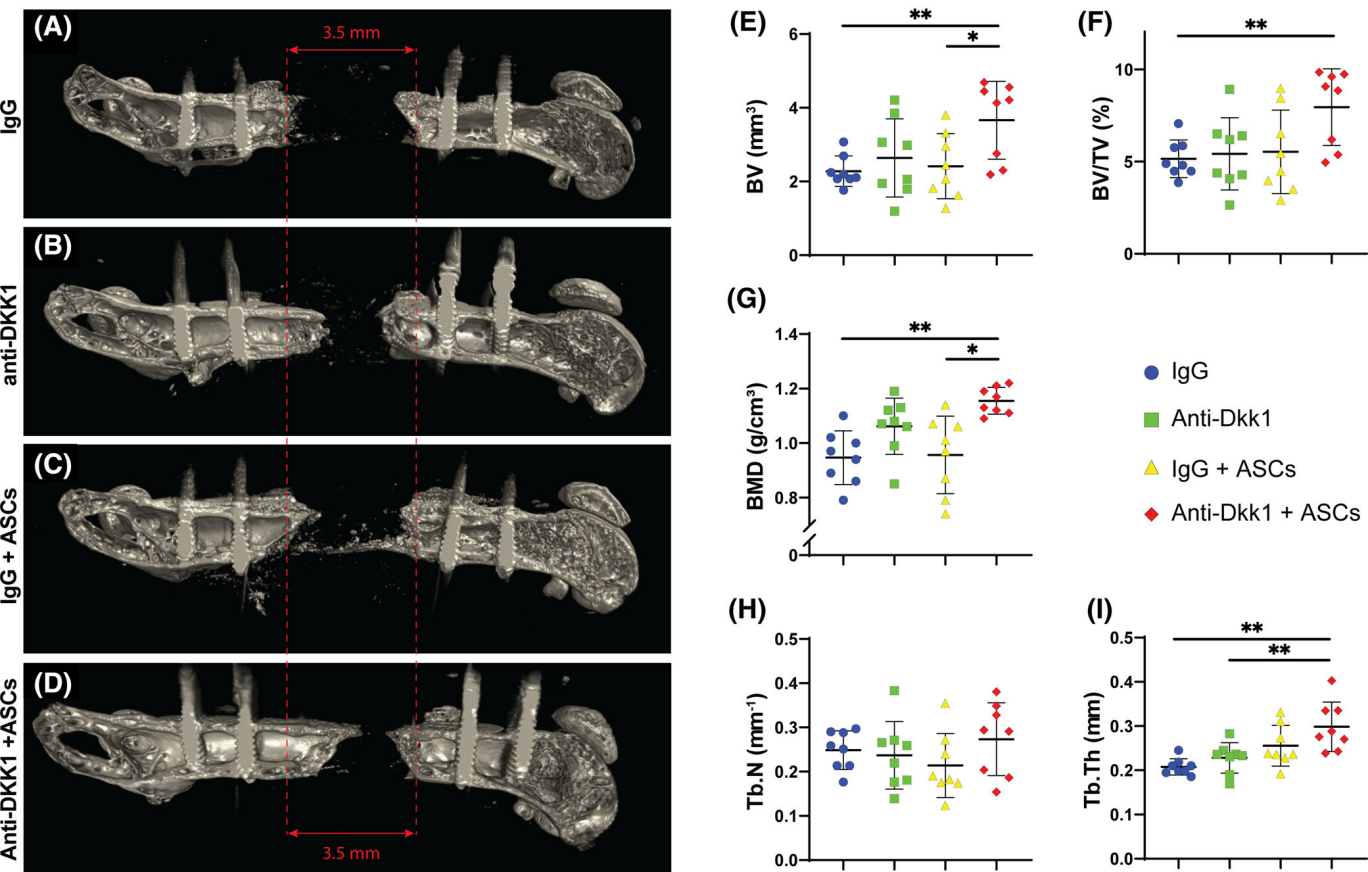
Anti‐DKK1 treatment incites ASC‐mediated bone formation as assessed by microcomputed tomography (μCT). Defects were treated with ASC seeded scaffolds or acellular control scaffolds. Animals were treated with anti‐DKK1 or IgG control (15 mg/kg, SC, twice weekly). A‐D, Representative three‐dimensional (3D) μCT reconstructions of the femoral segmental defect site at 8 weeks postoperative, shown from a sagittal perspective. Original defect size indicated by red lines. E‐I, Quantitative μCT analysis of the femoral segmental defect site, including (E) bone volume (BV), (F) fractional bone volume (BV/TV), (G) bone mineral density (BMD), (H) trabecular number (Tb.N), and (I) trabecular thickness (Tb.Th). Dots in scatterplots represent an individual animal, while crosshairs and whiskers represent mean and 1 SD, respectively. See Table S3 for a further summary of animal allocation, treatment regimens, and total cell numbers. *P < .05; **P < .01. ASCs, adipose‐derived stem cells

Bone formation within FSDs was next assessed using high‐resolution μCT imaging at the study endpoint (Figure 3). 3D sagittal μCT reconstructions of representative femurs confirmed the impression by XR, again showing an increase in bone formation among ASC treated defects with systemic anti‐DKK1 in comparison to other treatment groups (Figure 3A‐D). These radiographic findings were confirmed using quantitative μCT analysis across all animals, including analysis of BV, bone volume density (BV/TV), and BMD (Figure 3E‐G). Here, the combination anti‐DKK1 + ASCs treatment group resulted in a significant increase in all metrics in relation to IgG control (22.1%‐60.7% increase across measured parameters). Trabecular bone analysis showed an increase in trabecular thickness (Tb.Th), although no change in trabecular number (Tb.N) was observed (Figure 3H,I).

### 3.5 Anti‐DKK1 treatment enhances ASC‐mediated bone matrix formation

**FIGURE 4:**
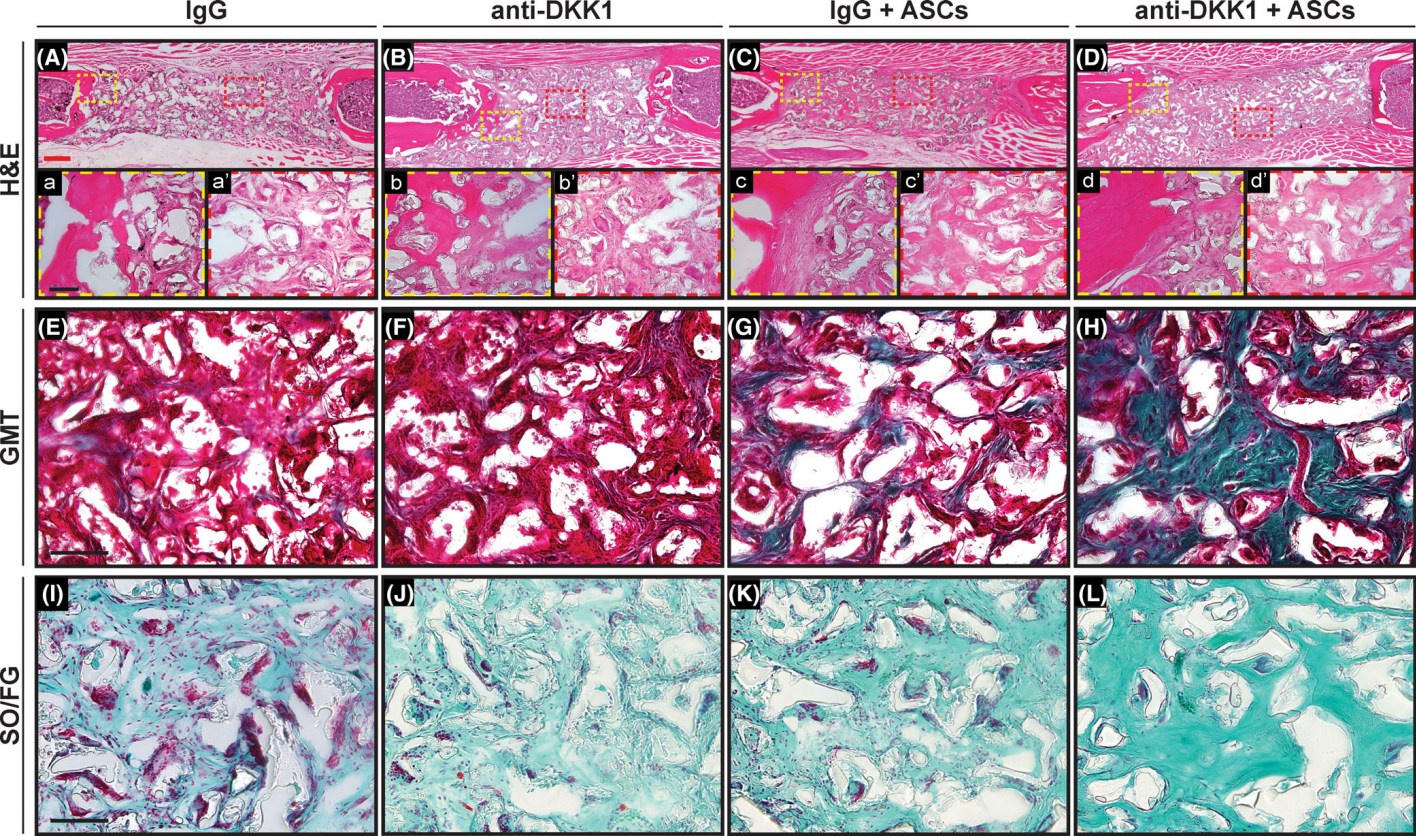
Anti‐DKK1 treatment induces ASC‐mediated bone matrix formation. Defects were treated with ASC seeded scaffolds or acellular control scaffolds. Animals were treated with anti‐DKK1 or IgG control (15 mg/kg, SC, twice weekly). A‐D, H&E staining among the entire defect span for each treatment group. High magnification (×20) of (a‐d) the bone‐scaffold interface and (a′‐d′) within the implant site. E‐H, Goldner's Modified Trichrome (GMT) staining among scaffold area of each group. Bone matrix appears blue/green, while fibrous tissue appears red. I‐L, Safranin O/Fast green (SO/FG) staining among scaffold area. Bone matrix appears darker green while cartilage (if present) would appear orange. All analyses performed at 8 weeks postimplantation. See Table S3 for a further summary of animal allocation and total cell numbers. Black scale bars = 50 μm; red scale bar = 400 μm

Histological analyses were next performed, which further confirmed morphologic differences associated with systemic anti‐DKK1 treatment of ASC treated bone defects (Figure 4). H&E staining among the whole scaffold area (Figure 4A‐D) showed bone matrix deposition on the edges of the defect in all the samples, while only the anti‐DKK1 + ASCs had robust bone matrix in the middle of the defect (Figure 4A′‐D′). GMT staining confimed this finding. Within the groups without ASCs therapy, no clear bone matrix deposition was found (Figure 4E,F). Immature bone formation was observed among the IgG + ASCs treatment group (Figure 4G), while prominent woven bone was observed among defect sites among the anti‐DKK1 + ASCs group. SO/FG staining was performed, which did not reveal any significant cartilaginous tissue among any treatment group (Figure 4I‐L).

### 3.6 Anti‐DKK1 promotes osteoblast differentiation and inhibits osteoclasts activity among FSDs

**FIGURE 5:**
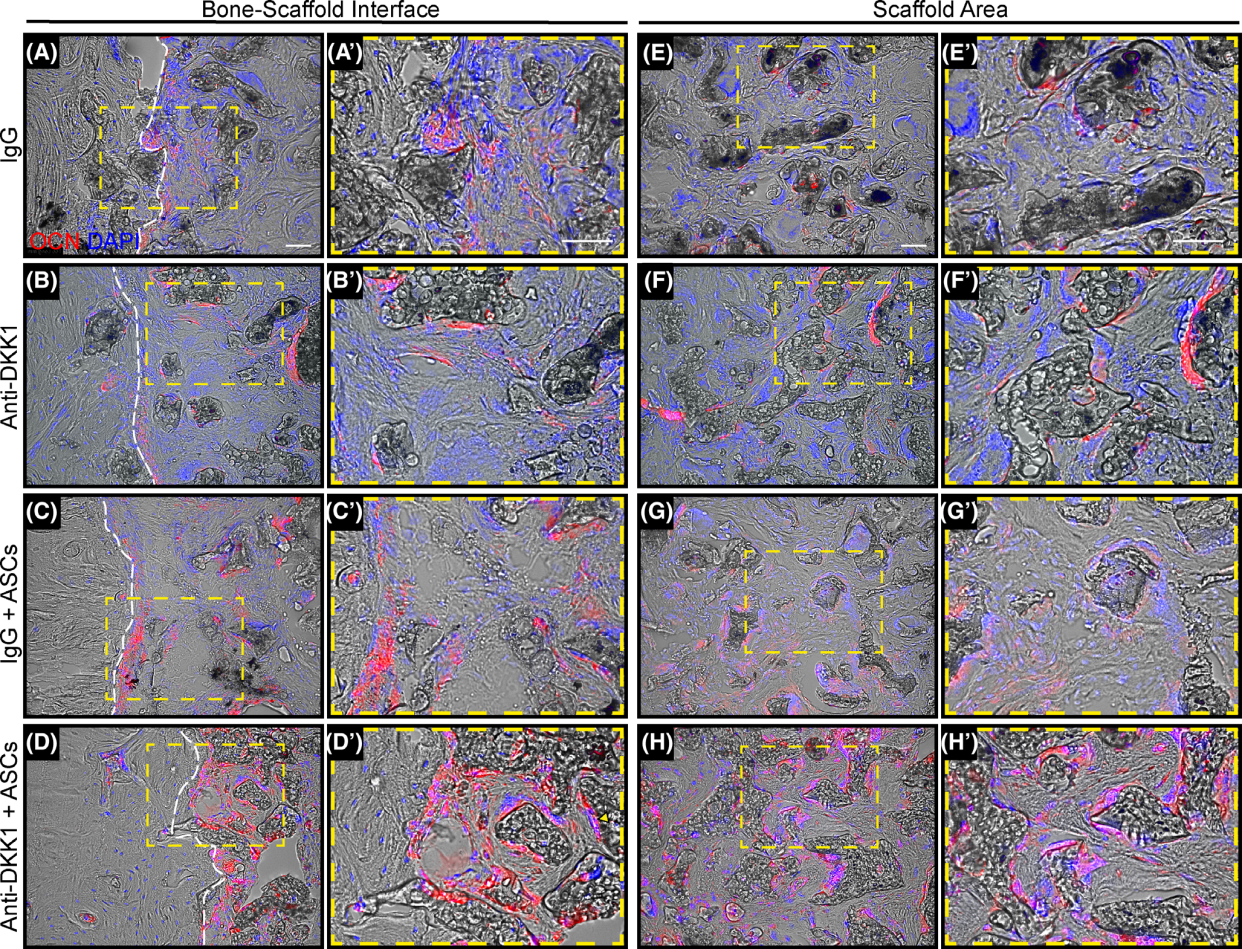
Anti‐DKK1 promotes osteoblast differentiation of ASCs and resident osteoprogenitor cells. Defects were treated with ASC seeded scaffolds or acellular control scaffolds. Animals were treated with anti‐DKK1 or IgG control (15 mg/kg, SC, twice weekly). Representative osteocalcin (OCN) immunohistochemical staining of the femoral segmental defects, either at the (A‐D) bone‐scaffold interface or (E‐H) within the implant site. A′‐H′, High magnification insets also shown. OCN appears red, while DAPI nuclear counterstain appears blue. White dashed lines outline the bone/scaffold interface. All analyses performed at 8 weeks postimplantation. See Table S3 for a further summary of animal allocation, treatment regimens, and total cell numbers. White Scale bars = 50 μm. ASCs, adipose‐derived stem cells. DAPI, 4′,6‐diamidino‐2‐phenylindole

The osteoblast specific marker osteocalcin (OCN) was next evaluated across the different groups using immunofluorescent staining (Figure 5), cross‐reactive with both mouse and human OCN proteins. Images were obtained either at the bone edge/scaffold interface (Figure 5A‐D) and within the central aspect of the scaffold areas (Figure 5E‐H). A conspicuous increase in OCN immunostaining was present among both ASC cell therapy groups, observable both within the bone‐scaffold interface and the central scaffold areas. Across all sections, ASC treated defects with anti‐DKK1 administration showed the most conspicuous OCN immunoreactivity.

Osteoclast activity was next assessed using TRAP stained sections (Figure S4). Representative images were again obtained from either the bone‐scaffold interface (Figure S4A‐D) or central scaffold area (Figure S4E‐H). In general, ASC treated bone defects demonstrated significantly higher TRAP staining than that of acellular controls. ASC treated defects with IgG control treatment showed the most conspicuous TRAP staining across samples. Consistent with known antiosteoclastic effects of anti‐DKK1,^36^, ^37^ the combination treatment anti‐DKK1 with ASCs showed a clear reduction in TRAP staining intensity and distribution in comparison to the IgG + ASCs treatment group.

### 3.7 Anti‐DKK1 increases ASC survival and enhances vascular ingrowth

**FIGURE 6:**
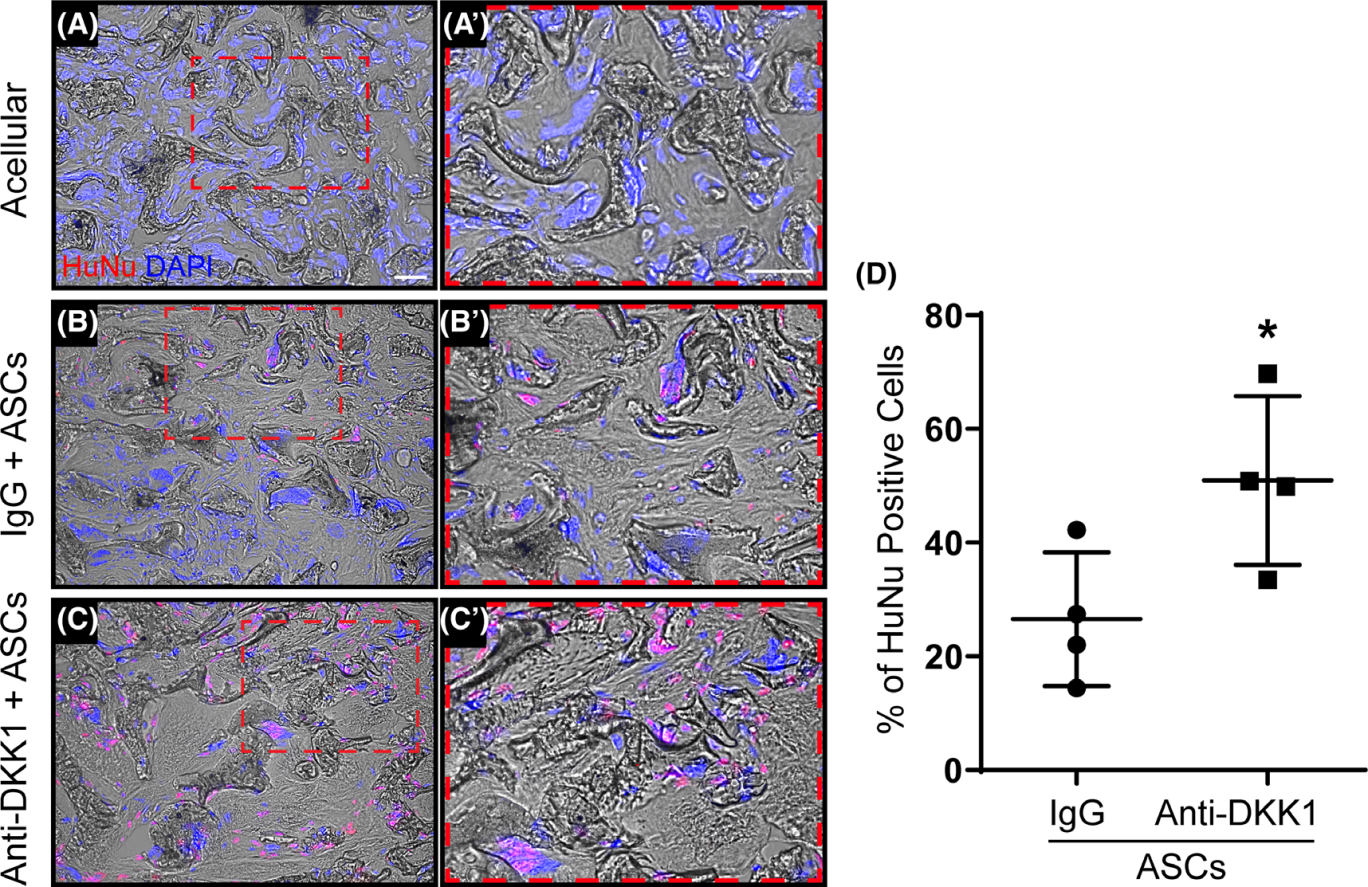
Anti‐DKK1 increases long‐term persistence of human ASCs within femoral segmental defects. Defects were treated with ASC seeded scaffolds or acellular control scaffolds. Animals were treated with anti‐DKK1 or IgG control (15 mg/kg, SC, twice weekly). A‐C, Immunofluorescence staining of human‐specific nuclei (HuNu) at 8 weeks postimplantation. A,A′, Acellular IgG control; B,B′, IgG + ASCs; C,C′, anti‐DKK1 + ASCs. Human cells appear red while DAPI nuclear counterstain appears blue. D, Quantification of HuNu immunohistochemical detection (% positive cells per random 10x field of view, n = 4 samples per group analyzed). White scale bars = 50 μm. *P < .05. ASCs, adipose‐derived stem cells. DAPI, 4′,6‐diamidino‐2‐phenylindole

Next, the potential effects of anti‐DKK1 on ASC persistence within FSD sites were examined, using immunofluorescent staining for human‐specific nuclei (HuNu) (Figure 6). Use of acellular treatment groups without human cell implantations confirmed the specificity of staining (Figure 6A). Systemic anti‐DKK1 treatment led to a clear increase in the number of residual human cells at the study endpoint, which were found most frequently lining woven bone (Figure 6B,C). Quantification of HuNu immunostaining demonstrated a 92% increase among anti‐DKK1 treated animals (Figure 6D) (*P < .05). Furthermore, co‐immunohistochemical stainings for HuNu and OCN was performed (Figure S5). These results showed a high degree of overlap between HuNu and OCN staining in the context of anti‐DKK1 treatment.

One potential mechanism for increased ASC engraftment and survival would be the direct upregulation of antiapoptotic gene expression among implanted ASCs by anti‐DKK1. An induction of such genes, such as BCL2 and MCL1, has been reported by anti‐DKK1 in other cell types.^38^ Targeted antiapoptotic genes were investigated by qPCR under in vitro conditions with anti‐DKK1 treatment vs IgG control (Figure S6A‐C). Both *BCL2* and *BCL2A1* were significantly upregulated after anti‐DKK1 treatment (Figure S6A,B, *P < .01) while *MCL1* expression was not significantly affected (Figure S6C).

**FIGURE 7:**
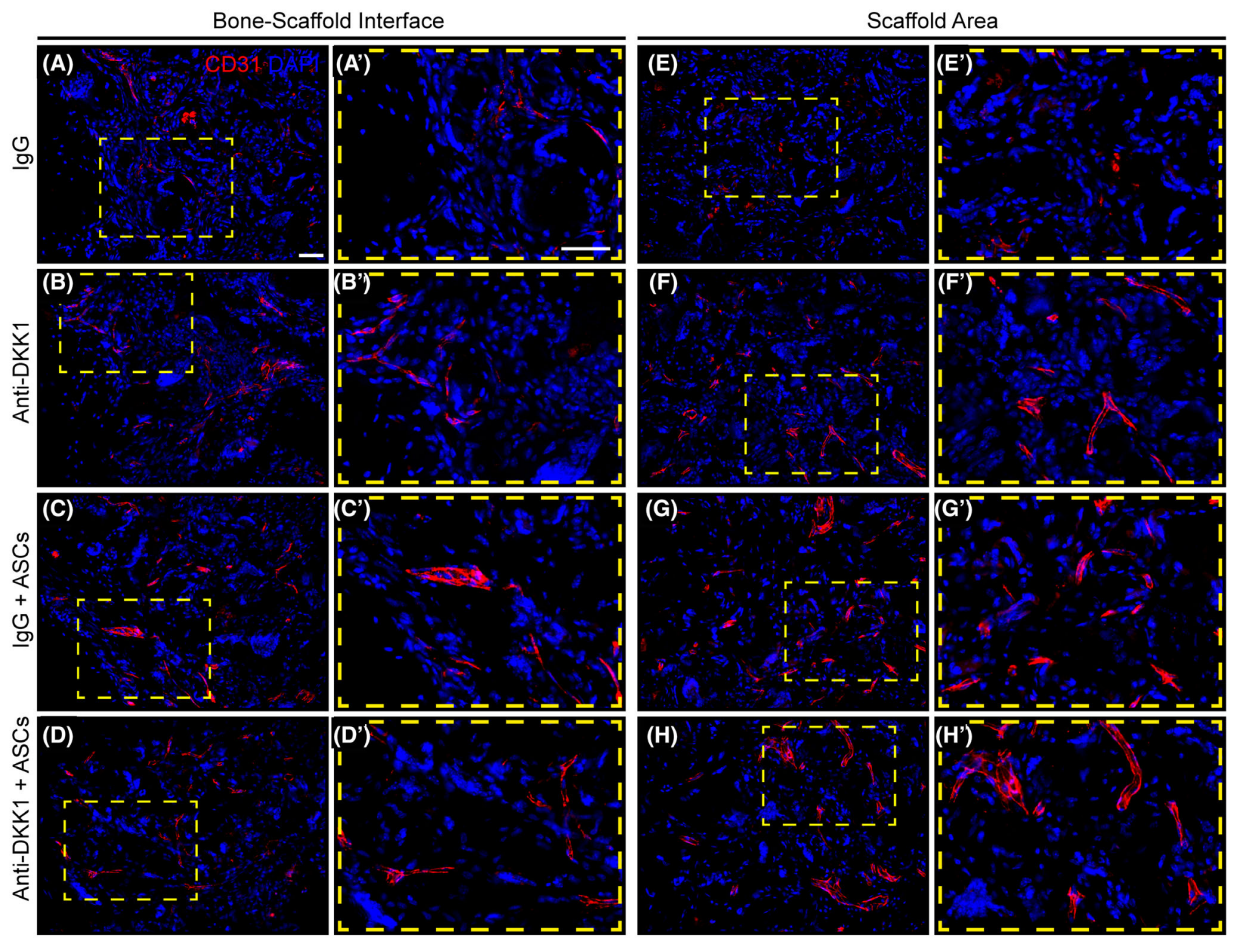
Vascular ingrowth is promoted by both ASC therapy and DKK1 neutralization. Defects were treated with ASC seeded scaffolds or acellular control scaffolds. Animals were treated with anti‐DKK1 or IgG control (15 mg/kg, SC, twice weekly). CD31 immunohistochemical staining of bone‐scaffold interface (A‐E), and within the implant site (E‐H). High magnification insets show in detail of vascular distribution (A′‐H′). CD31^+^ endothelial cells appear red, while DAPI nuclear counterstain appears blue. All analyses performed at 8 weeks postimplantation. White scale bars = 50 μm. ASCs, adipose‐derived stem cells. DAPI, 4′,6‐diamidino‐2‐phenylindole

Another potential mechanism for increased ASC engraftment and survival could be increased vascular ingrowth. To assess this, CD31 immunohistochemical staining was performed across defect sites (Figure 7). Images were again obtained either at the bone‐scaffold interface (Figure 7A‐D) or central scaffold area (Figure 7E‐H). A paucity of CD31^+^ vascular channels was observed among IgG acellular control defects. In contrast, all three interventions led to an increase in defect site vascularity. Both ASC treatment groups showed the highest frequency of vascular ingrowth, although no clear differences were found between IgG + ASC and anti‐DKK1 + ASC treatment groups.

### 3.8 Anti‐DKK1 promotes osseointegration

Defect fixation required internal stabilization with four titanium screws. While osseous integration of the fixation system was not the primary intent of the study, a conspicuous increase in peri‐screw bone formation was observed in some samples during our analyses. This prompted analysis of osseous integration across treatment groups (Figure S7). Using established methods for quantification of bone around a metal implant, a volume of interest was constructed around each screw, and mean BV calculated per animal and treatment group. A clear increase in peri‐screw bone formation was seen among all anti‐DKK1 treated animals, by both μCT cross sectional images (Figure S7A‐D) and H&E staining (Figure S7A′‐D′). Quantitative μCT assessment confirmed these impressions, and revealed a significant increase in BV among anti‐DKK1 treated groups (Figure S7E, 18%‐34% increase across anti‐DKK1 treatment groups, **P < .01).

## 4 DISCUSSION

Adipose‐derived therapies have potential use for cell‐augmented bone repair strategies.^4^, ^5^, ^6^, ^7^, ^8^, ^39^ Yet, inconsistent repair results in bone tissue engineering^40^, ^41^ have been linked to cell population heterogeneity, variability in cell preparation, or expression of osteogenic differentiation inhibitors.^11^, ^13^ Our observations suggest that DKK1 inhibits the early osteogenic differentiation of human ASCs,^26^ and that systemic anti‐DKK1 therapy has benefit for the ASC engraftment, survival, and osteodifferentiation, associated with improved defect site revascularization and eventual reossification. In this regard, anti‐DKK1 is one of only several systemic drugs which has been shown to improve stem cell‐augmented bone repair, which also includes intermittent Parathyroid hormone (PTH).^42^

One remarkable finding is a near double in the persistence of engrafted human Adipose‐derived stem cells (hASC) with systemic anti‐DKK1 treatment. Poor survival of autologous or allogeneic stem cells has been one feature cited as a limitation to current efforts in cell‐augmented bone repair.^30^ In prior studies, anti‐DKK1 led to a modest increase in proliferation of hASCs which would unlikely result in this large scale change in cell persistence within a bone defect.^26^ Instead, neutralization of DKK1 may function to prevent cell death within the engrafted hASC population. A large body of literature in other neoplastic and non‐neoplastic cell types suggests that DKK1 induces apoptosis, such as in epithelial neoplasms,^43^ in limb development^44^ and in instances of cartilage degeneration with arthritis.^38^ It is likely that analogous findings were present in our stem cell xenograft model, in which systemic DKK1 led to a reduction in hASC apoptosis.

It is intriguing to speculate if this increase in stem cell survival is also related to vascular ingrowth. The majority of evidence shows that DKK1 inhibits in vitro and in vivo neoangiogenesis.^45^, ^46^ Reports indicate that this antiangiogenetic effect is by means of direct inhibition of endothelial cell proliferation and potentially by competitive binding of DKK2 to LRP6.^45^ Here we found that anti‐DKK1 conspicuously increased the vascularity among acellular groups, but did not obviously change vascular ingrowth between the two cell‐treated groups. More detailed studies must be performed in order to determine the relative contribution of increased vascular ingrowth to ASC survival within the early bone defect niche.

There are several limitations to the present study. First, to ensure consistent findings across animals, the in vivo experiment was performed with cells from a single female donor. Evidence suggests that ASC osteogenic differentiation potential differs on the basis of gender and location.^47^ Further study must confirm the broader applicability of these results to human ASCs from donors with different demographics. Second, our observations regarding the benefit of anti‐DKK1 in bone repair were observed in young, adult male animals. It will be interesting to determine the extent to which this result should be true or even more prominent in animals with low bone mass or advanced age.

## 5 CONCLUSION

Systemic anti‐DKK1 induces an increase in human ASC engraftment and survival, an increase in vascular ingrowth, and ultimately improved bone repair outcomes. These results suggest that anti‐DKK1 can be used as a method to augment cell‐mediated bone regeneration. Such an approach could be particularly valuable in the contexts of impaired bone healing, such as in osteoporotic bone repair.

## CONFLICT OF INTEREST

A.W.J. is a paid consultant for Novadip, and receives funding for unrelated research from MTF Biologics and Novadip. This arrangement has been reviewed and approved by the Johns Hopkins University in accordance with its conflict of interest policies. All of the other authors declared no potential conflicts of interest.

## AUTHOR CONTRIBUTIONS

S.N., Y.W.: collection and/or assembly of data, data analysis and interpretation, manuscript writing; T.S., Q.Q., G.C.‐Y.H., J.X., S.L., M.C., R.J.T., V.Y., A.P., C.A.M.: collection and/or assembly of data, data analysis and interpretation; K.B., M.L.: provision of study materials; A.W.J.: conception and design, manuscript writing, financial support, final approval of manuscript.

## Supporting information


**Supplementary Table S1** Supporting information

**Supplementary Figure S1** **In vitro assessment of hASC survival after anti‐DKK1 treatment and seeding on HA‐PLGA scaffolds.** (A, B) Live‐dead staining of ASC seeded scaffolds after 7d culture with anti‐DKK1 (2 μg/mL) or IgG isotype treatment. Live cells appear green while dead cells are red. (C) Quantification of (A, B) calculated as % cell viability among total cells per view. Scale bar: 50 μm.

**Supplementary Figure S2** **Anti‐DKK1 does not significantly affect hASC attachment.** Quantification of unattached hASCs among total seeded cells at 6 hours post seeding on HA‐PLGA scaffolds (2 μg/mL anti‐DKK1 or IgG isotype control).

**Supplementary Figure S3** **Anti‐DKK1 enhances mineralization of ASCs on HA‐PLGA scaffolds in vitro.** (A) Alizarin red staining of ASC‐scaffold complex after 7 days of osteogenic differentiation (2 μg/mL anti‐DKK1 or IgG isotype control). (B) Quantification of (A).

**Supplementary Figure S4** **Anti‐DKK1 inhibits osteoclasts activity among ASC‐treated femoral segmental defects.** Defects were treated with ASC seeded scaffolds or acellular control scaffolds. Animals were treated with anti‐DKK1 or IgG control (15 mg/kg, sc, twice weekly). Tartrate resistant acid phosphatase (TRAP) staining of the bone‐scaffold interface (A‐E), and within the implant site (E‐H). High magnification insets are shown (A'‐H′). TRAP positive areas appear purple while fast green acts as counterstain. All analyses performed at 8 weeks post‐implantation. Black scale bars: 40 μm

**Supplementary Figure S5** **Anti‐DKK1 enhances the osteogenic differentiation of human ASCs once implanted. (A, B)** Co‐immunohistochemical staining of human specific nuclei (HuNu) and Osteocalcin (OCN), assessed at 8 weeks post‐implantation. Human nuclei positive cells appear in green while OCN^+^ cells appear red. Scale bar: 50 μm

**Supplementary Figure S6** **Anti‐DKK1 induces anti‐apoptotic gene expression in hASCs.** Anti‐apoptotic gene expression after 3 days of anti‐DKK1 treatment (2 μg/mL) assessed by qRT‐PCR, including (A) *BCL2* (B‐Cell CLL/Lymphoma gene 2), (B) *BCL2A1* (BCL2 related protein A1), and (C) *MCL1* (Myeloid cell leukemia sequence 1). *P < 0.01.

**Supplementary Figure S7** **Systemic anti‐DKK1 promotes osseointegration of mechanical implants.** Representative axial (A‐D) and sagittal (A'‐D′) μCT cross‐sectional images of the bone / screw interface. The yellow lines describe the Region of Interest (ROI) used for the periscrew bone tissue quantification. (E‐H) Representative sagittal H&E stained sections of bone surrounding the screws after their removal. (E) Quantitative μCT analysis of bone volume surrounding the mechanical implant. Graphs represent mean and error bars represent one SD. Each dot represents the average bone volume value per animal. All analyses performed at 8 weeks post‐implantation. **P < 0.01.

## Data Availability

The data that support the findings of this study are available on request from the corresponding author.
