# Supplementary material for: Systemic DKK1 neutralization enhances human adipose‐derived stem cell mediated bone repair
Source: Stem Cells Transl Med. 2020 Dec 30;10(4):610–22. doi: 10.1002/sctm.20-0293 (PMC7980212; doi:10.1002/sctm.20-0293)
Supplement: Supplementary file 1 — Supplementary Table S1 Supporting information [file SCT3-10-610-s001.docx]

**Supplementary Materials**

**Supplementary Tables**

**Supplementary Table S1. Antibodies used**

| **Antibody** | **Company** | **Catalog #** | **Use** |
| --- | --- | --- | --- |
| Anti-DKK1 | R&D systems | AF1096 | N |
| Goat IgG | R&D systems | AB-108-C | N |
| Anti-Osteocalcin (OCN) | Abcam | ab93876 | IF (1:100) |
| Anti-Human Nuclei (HuNu) | Sigma | MAB1281 | IF ( 1:80 ) |
| Anti-CD31 | Abcam | ab28364 | IF (1:150) |
| Goat anti-Rabbit AF647 | Abcam | ab150079 | IF (1:200) |
| Goat anti-Mouse AF647 | Abcam | ab150119 | IF (1:200) |
| Goat anti-Rabbit DyLight 594 | Vector Laboratories | DI-1594 | IF (1:200) |
| N: Neutralization; IF: Immunofluorescent staining. | | | |

**Supplementary Table S2. Quantitative PCR primers used**

| **Gene** | **Forward** | **Reverse** |
| --- | --- | --- |
| *ALP* | ACCACCACGAGAGTGAACCA | CGTTGTCTGAGTACCAGTCCC |
| *AXIN2* | CAACACCAGGCGGAACGAA | GCCCAATAAGGAGTGTAAGGACT |
| *CCND1* | GCTGCGAAGTGGAAACCATC | CCTCCTTCTGCACACATTTGAA |
| *COL1A1* | GAGGGCCAAGACGAAGACATC | CAGATCACGTCATCGCACAAC |
| *DKK1* | ATAGCACCTTGGATGGGTATTCC | CTGATGACCGGAGACAAACAG |
| *GAPDH* | CTGGGCTACACTGAGCACC | AAGTGGTCGTTGAGGGCAATG |
| *RUNX2* | TGGTTACTGTCATGGCGGGTA | TCTCAGATCGTTGAACCTTGCTA |
| *WNT5A* | ATTCTTGGTGGTCGCTAGGTA | CGCCTTCTCCGATGTACTGC |
| *BCL2* | TTGTTCAAACGGGATTCACA | GAGCAAGTGCAGCCACAATA |
| *MCL1* | TGCTTCGGAAACTGGACATCA | TAGCCACAAAGGCACCAAAAG |
| *BCL2A1* | TACAGGCTGGCTCAGGACTAT | CGCAACATTTTGTAGCACTCTG |

**Supplementary Table S3. Description of the experimental groups**

| **Group** | **Systemic Treatment (15 mg/kg, SC, twice weekly over 4 weeks)** | **Cell #/Implant** | **Final**  **Timepoint** | **Animal #** |
| --- | --- | --- | --- | --- |
| IgG | IgG isotype | - | 8 weeks | 8 |
| Anti-Dkk1 | Anti-Dkk1 | - | 8 weeks | 8 |
| IgG + ASCs | IgG isotype | 7.5 x 10^5^ | 8 weeks | 8 |
| Anti-Dkk1 + ASCs | Anti-Dkk1 | 7.5 x 10^5^ | 8 weeks | 8 |

**Supplementary Table S4. Changes in Bone Mineral Density (BMD) with systemic anti-Dkk1 (mean ± SD), assessed by DXA**

|  | **BMD (mg/cm^2^)** | | | |
| --- | --- | --- | --- | --- |
|  | **IgG** | **Anti-Dkk1** | **ASCs + IgG** | **ASCs + Anti-Dkk1** |
| **Contralateral Femur** |  |  |  |  |
| 0 wks | 117.1 ± 10.35 | 113.8 ± 12.82 | 122.5 ± 13.68 | 113.8 ± 12.82 |
| 4 wks | 112.7 ± 7.85 | 115.7 ± 18.92 | 121.3 ± 9.65 | 115.7 ± 18.92 |
| 8 wks | 109.1 ± 9.76 | 112.9 ± 9.12 | 119.3 ± 8.01 | 112.9 ± 9.12 |
| **L1-L6** |  |  |  |  |
| 0 wks | 74.43 ± 4.74 | 81.17 ± 15.04 | 77.88 ± 5.75 | 91.69 ± 16.92 |
| 4 wks | 73.87 ± 6.29 | 89.23 ± 35.58 | 78.11 ± 8.30 | 83.76 ± 8.20 |
| 8 wks | 69.94 ± 7.88 | 75.04 ± 13.62 | 74.15 ± 7.13 | 90.76 ± 14.41 |
