# Supplementary figures and images for: Systemic DKK1 neutralization enhances human adipose‐derived stem cell mediated bone repair
Source: Stem Cells Transl Med. 2020 Dec 30;10(4):610–22. doi: 10.1002/sctm.20-0293 (PMC7980212; doi:10.1002/sctm.20-0293)

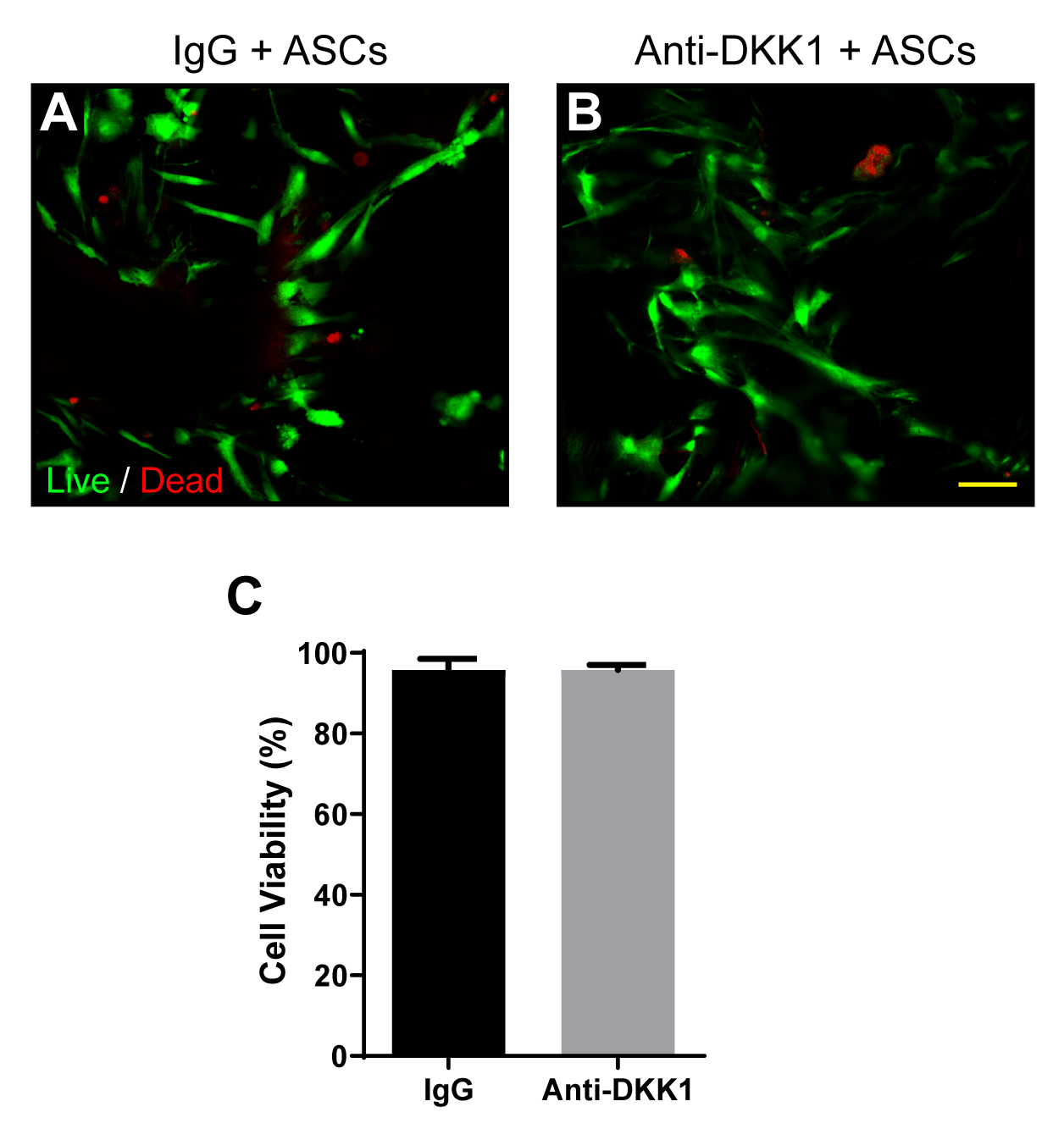

Supplement: Supplementary file 2 — Supplementary Figure S1 In vitro assessment of hASC survival after anti‐DKK1 treatment and seeding on HA‐PLGA scaffolds. (A, B) Live‐dead staining of ASC seeded scaffolds after 7d culture with anti‐DKK1 (2 μg/mL) or IgG isotype treatment. Live cells appear green while dead cells are red. (C) Quantification of (A, B) calculated as % cell viability among total cells per view. Scale bar: 50 μm. [file SCT3-10-610-s006.tif]

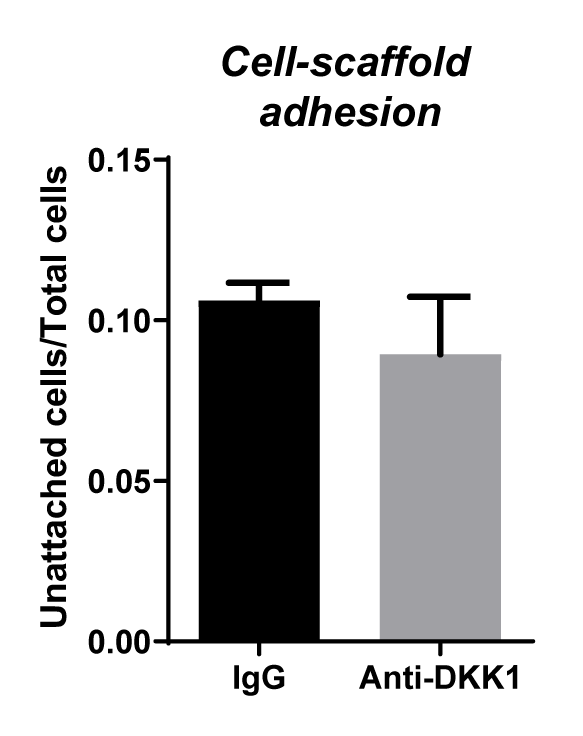

Supplement: Supplementary file 3 — Supplementary Figure S2 Anti‐DKK1 does not significantly affect hASC attachment. Quantification of unattached hASCs among total seeded cells at 6 hours post seeding on HA‐PLGA scaffolds (2 μg/mL anti‐DKK1 or IgG isotype control). [file SCT3-10-610-s007.tif]

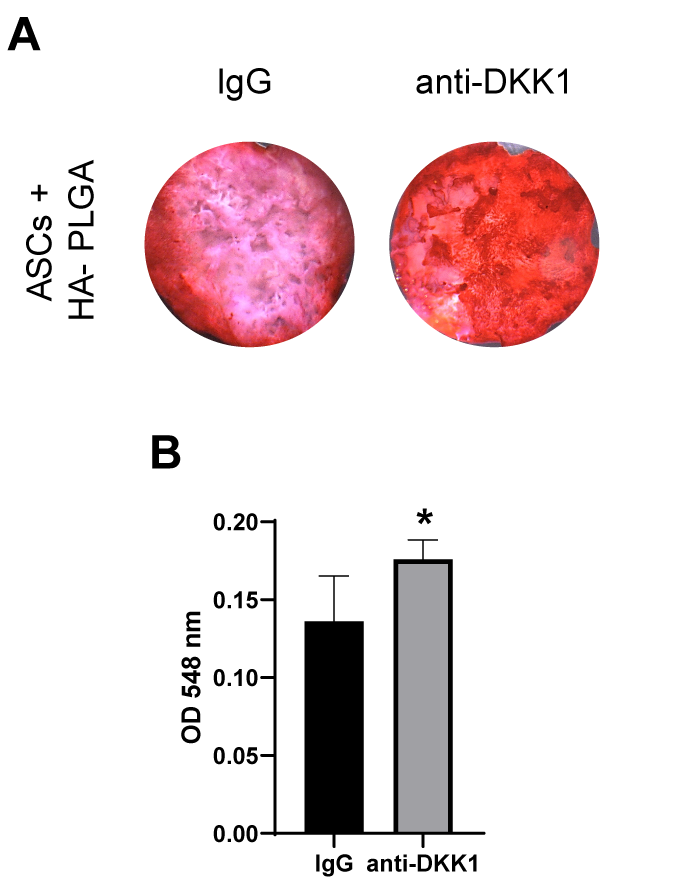

Supplement: Supplementary file 4 — Supplementary Figure S3 Anti‐DKK1 enhances mineralization of ASCs on HA‐PLGA scaffolds in vitro. (A) Alizarin red staining of ASC‐scaffold complex after 7 days of osteogenic differentiation (2 μg/mL anti‐DKK1 or IgG isotype control). (B) Quantification of (A). [file SCT3-10-610-s005.tif]

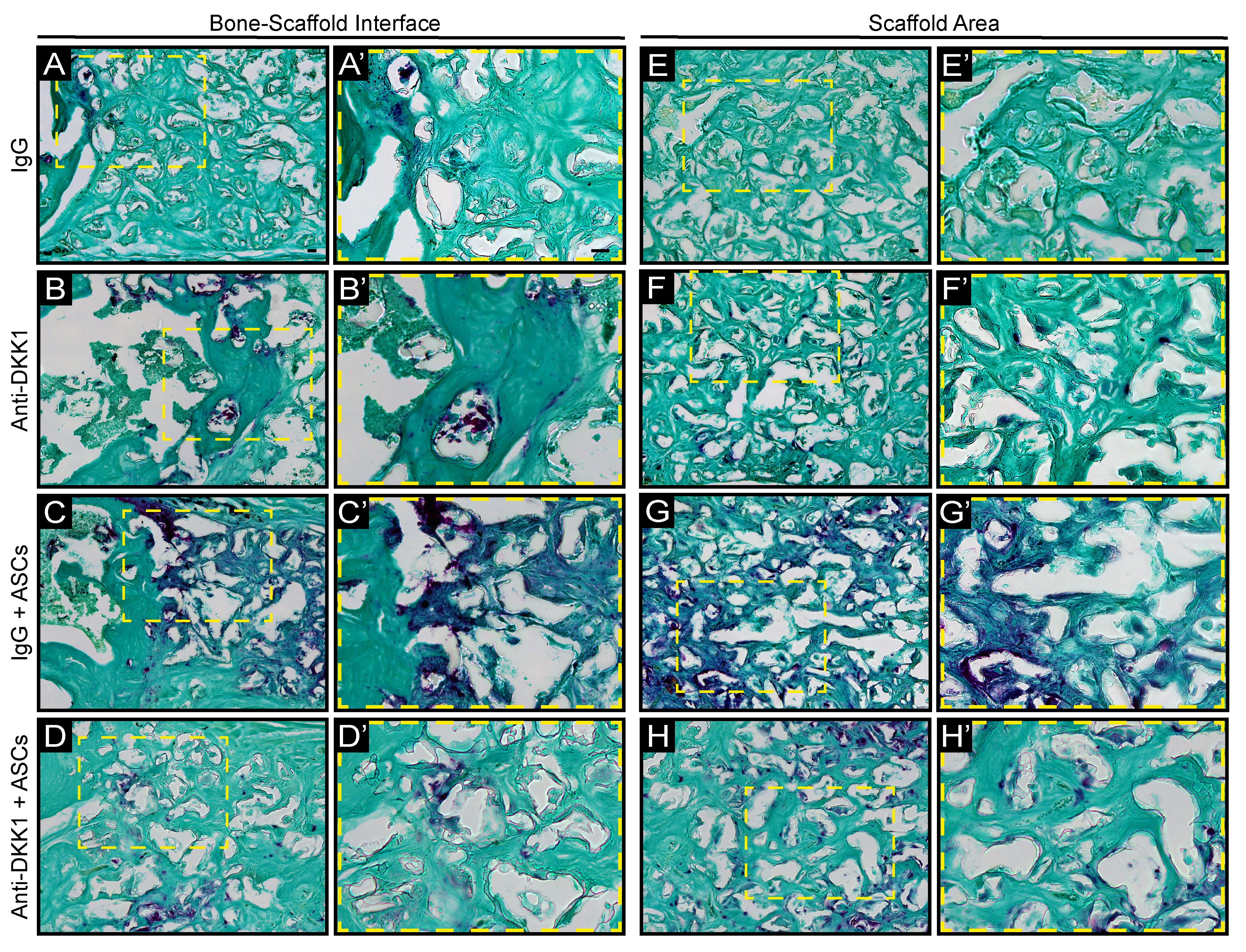

Supplement: Supplementary file 5 — Supplementary Figure S4 Anti‐DKK1 inhibits osteoclasts activity among ASC‐treated femoral segmental defects. Defects were treated with ASC seeded scaffolds or acellular control scaffolds. Animals were treated with anti‐DKK1 or IgG control (15 mg/kg, sc, twice weekly). Tartrate resistant acid phosphatase (TRAP) staining of the bone‐scaffold interface (A‐E), and within the implant site (E‐H). High magnification insets are shown (A'‐H′). TRAP positive areas appear purple while fast green acts as counterstain. All analyses performed at 8 weeks post‐implantation. Black scale bars: 40 μm [file SCT3-10-610-s004.tif]

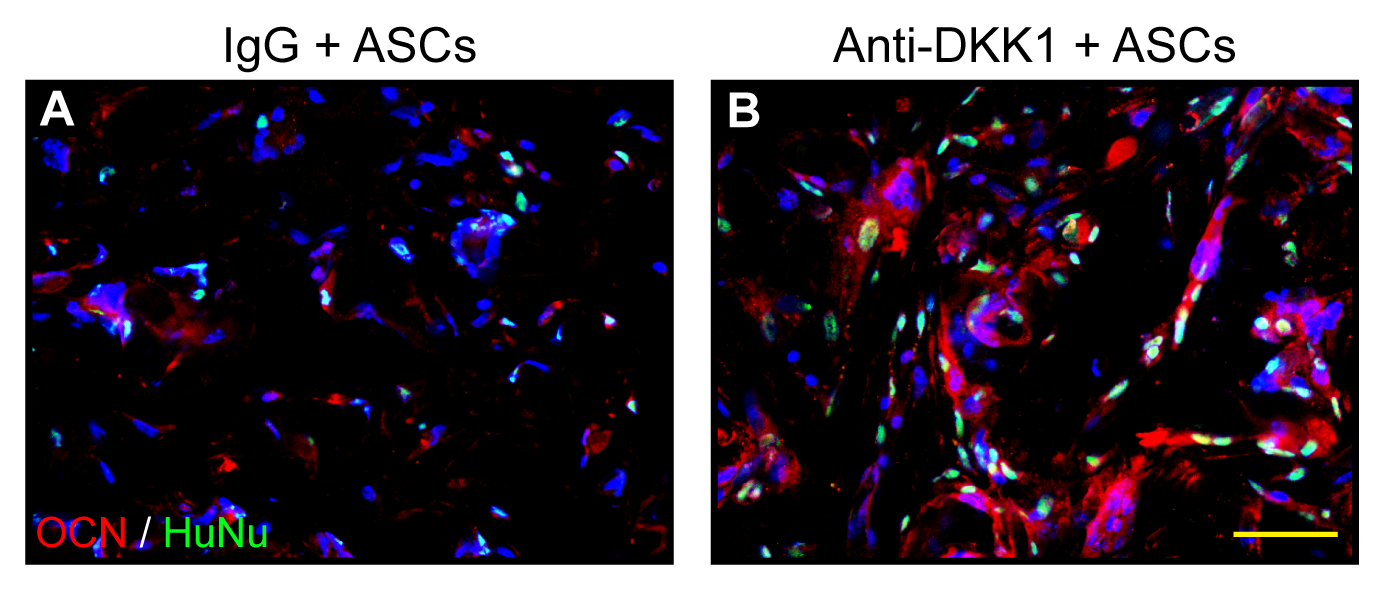

Supplement: Supplementary file 6 — Supplementary Figure S5 Anti‐DKK1 enhances the osteogenic differentiation of human ASCs once implanted. (A, B) Co‐immunohistochemical staining of human specific nuclei (HuNu) and Osteocalcin (OCN), assessed at 8 weeks post‐implantation. Human nuclei positive cells appear in green while OCN+ cells appear red. Scale bar: 50 μm [file SCT3-10-610-s003.tif]

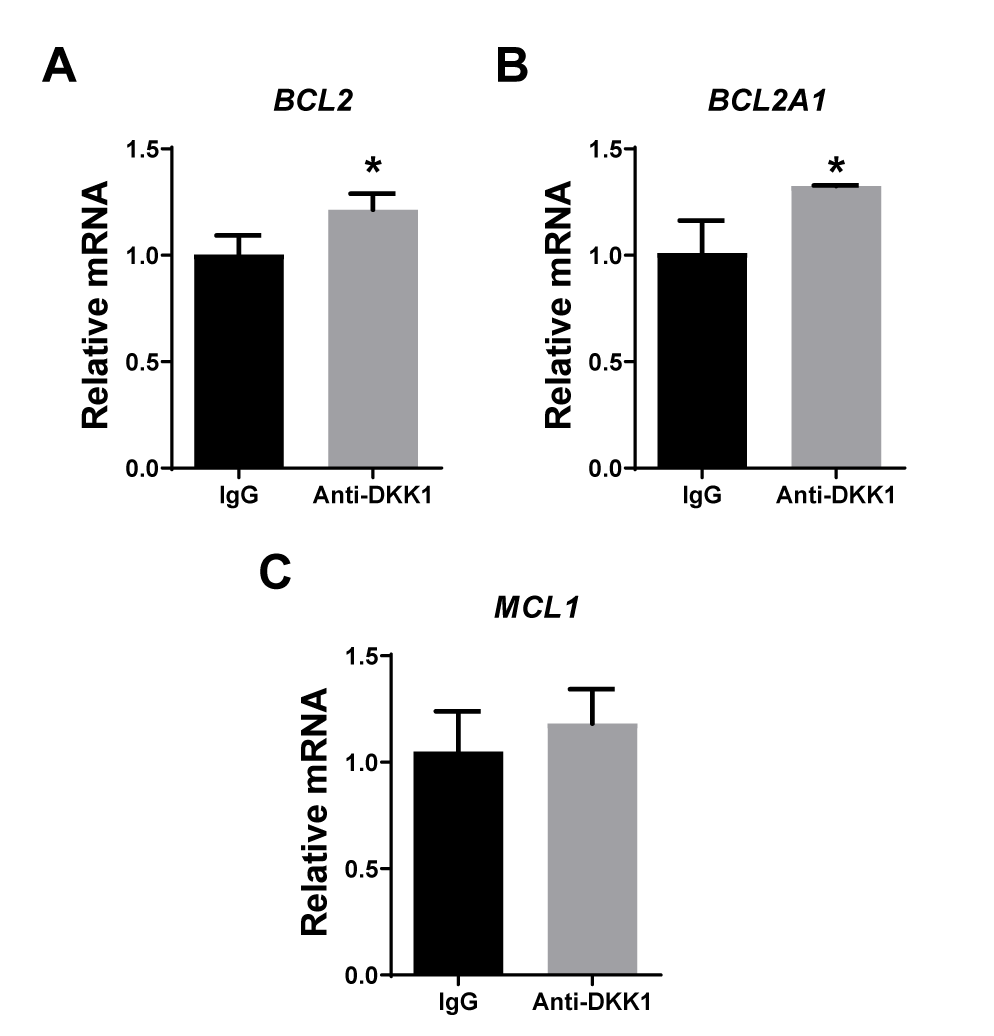

Supplement: Supplementary file 7 — Supplementary Figure S6 Anti‐DKK1 induces anti‐apoptotic gene expression in hASCs. Anti‐apoptotic gene expression after 3 days of anti‐DKK1 treatment (2 μg/mL) assessed by qRT‐PCR, including (A) BCL2 (B‐Cell CLL/Lymphoma gene 2), (B) BCL2A1 (BCL2 related protein A1), and (C) MCL1 (Myeloid cell leukemia sequence 1). *P < 0.01. [file SCT3-10-610-s002.tif]

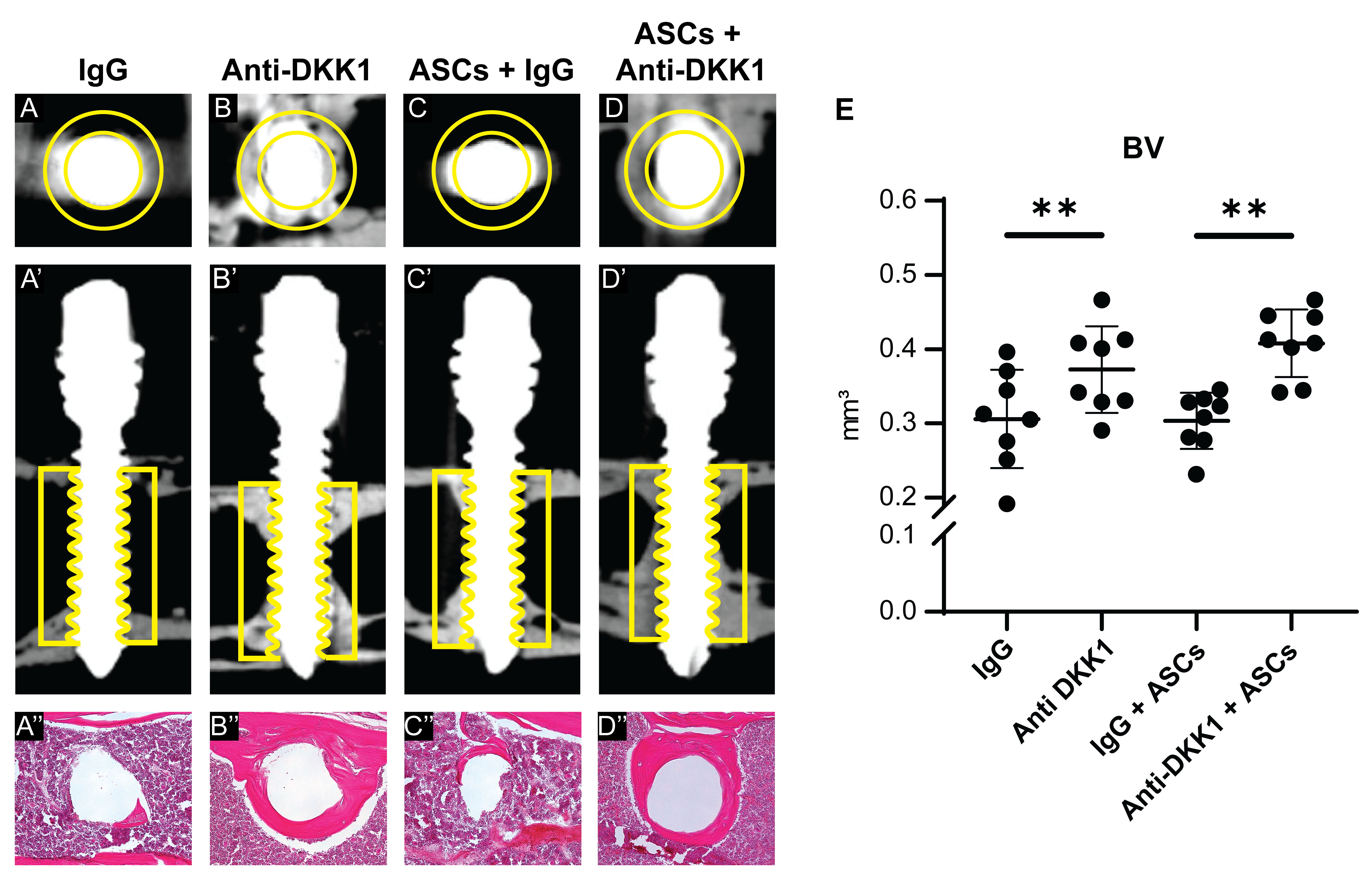

Supplement: Supplementary file 8 — Supplementary Figure S7 Systemic anti‐DKK1 promotes osseointegration of mechanical implants. Representative axial (A‐D) and sagittal (A'‐D′) μCT cross‐sectional images of the bone / screw interface. The yellow lines describe the Region of Interest (ROI) used for the periscrew bone tissue quantification. (E‐H) Representative sagittal H&E stained sections of bone surrounding the screws after their removal. (E) Quantitative μCT analysis of bone volume surrounding the mechanical implant. Graphs represent mean and error bars represent one SD. Each dot represents the average bone volume value per animal. All analyses performed at 8 weeks post‐implantation. **P < 0.01. [file SCT3-10-610-s008.tif]
